# Formation of Asymmetrical Structured Silica Controlled by a Phase Separation Process and Implication for Biosilicification

**DOI:** 10.1371/journal.pone.0061164

**Published:** 2013-04-09

**Authors:** Jia-Yuan Shi, Qi-Zhi Yao, Xi-Ming Li, Gen-Tao Zhou, Sheng-Quan Fu

**Affiliations:** 1 Key Laboratory of Crust-Mantle Materials and Environments, Chinese Academy of Sciences, School of Earth and Space Sciences, University of Science and Technology of China, Hefei, People's Republic of China; 2 School of Chemistry and Materials, University of Science and Technology of China, Hefei, People's Republic of China; 3 Hefei National Laboratory for Physical Sciences at Microscale, University of Science and Technology of China, Hefei, People's Republic of China; RMIT University, Australia

## Abstract

Biogenetic silica displays intricate patterns assembling from nano- to microsize level and interesting non-spherical structures differentiating in specific directions. Several model systems have been proposed to explain the formation of biosilica nanostructures. Of them, phase separation based on the physicochemical properties of organic amines was considered to be responsible for the pattern formation of biosilica. In this paper, using tetraethyl orthosilicate (TEOS, Si(OCH_2_CH_3_)_4_) as silica precursor, phospholipid (PL) and dodecylamine (DA) were introduced to initiate phase separation of organic components and influence silica precipitation. Morphology, structure and composition of the mineralized products were characterized using a range of techniques including field emission scanning electron microscopy (FESEM), transmission electron microscope (TEM), X-ray diffraction (XRD), thermogravimetric and differential thermal analysis (TG-DTA), infrared spectra (IR), and nitrogen physisorption. The results demonstrate that the phase separation process of the organic components leads to the formation of asymmetrically non-spherical silica structures, and the aspect ratios of the asymmetrical structures can be well controlled by varying the concentration of PL and DA. On the basis of the time-dependent experiments, a tentative mechanism is also proposed to illustrate the asymmetrical morphogenesis. Therefore, our results imply that in addition to explaining the hierarchical porous nanopatterning of biosilica, the phase separation process may also be responsible for the growth differentiation of siliceous structures in specific directions. Because organic amine (e.g., long-chair polyamines), phospholipids (e.g., silicalemma) and the phase separation process are associated with the biosilicification of diatoms, our results may provide a new insight into the mechanism of biosilicification.

## Introduction

Biomineralization is the formation of hard tissues with complex structures and multifunctional properties, which occurs in almost all the living organisms from prokaryotes to humans [1], [2]. Some of the morphologically gorgeous and structurally intricate biominerals are exemplified by the biosilica formed in the aquatic organisms including diatoms and sponges [3], [4]. These biogenic minerals are structured in the nanometer to micrometer scale range, and composed of amorphous silica [5]–[7].

Diatom is well known for the spectacular design of its silica-based cell wall (termed frustules) [2], [8], [9]. More than 40 years ago, Nakajima and Volcani have noticed that diatom biosilica contained unusual amino acid derivatives such as N,N,N-trimethylhydroxylysine and dihydroxyproline [10], [11]. This observation is the first to indicate that diatom silica is a composite material. In recent decades, a variety of organic and biological molecules have been successfully separated and identified from cell-wall extracts of diatoms[12], [13]. An emerging consensus is that polysaccharides [14], [15], proteins [16]–[20], and polyamines [21] are general organic components of diatom cell walls. In such a context, many efforts have been made to explore how these components interact with silicic acid, silicate, or silicon-containing compound, and influence silica morphogenesis [2], [16], [22], [23].

In terms of polyamines, all genera of diatoms investigated so far incorporate polyamines into their silica-based cell walls [24]. Most surprisingly, cell-wall extracts from *Coscinodiscus* diatoms exhibit predominately polyamines, whereas silaffin-related peptides appear to be absent [25]. These observations stimulate a polyamines-based phase separation model to be proposed for the pattern formation of the diatom cell-walls with hierarchically hexagonal porous structures [25]. In this model, polyamines are able to undergo a phase-separation process within a specialized membrane-bound compartment termed silica deposition vesicle to form an emulsion of microdroplets. These droplets form a hexagonally arranged monolayer within the silica deposition vesicle. Silica precipitation occurs at the interface between the solution and the organic microdroplets [26], which cause the formation of honeycomb-like framework. A defined fraction of the polyamine population is consumed by its co-precipitation with silica. As a result, smaller droplets separate from the surface of the original microdroplet. Silicification continues at these newly created water/polyamine interfaces of smaller droplets and a smaller hexagonal package of silica is thereupon developed. This mechanism would allow the creation of additional hexagonal frameworks at smaller and smaller scales. Finally, hierarchically porous structures and spectacular patterns are exhibited in the silica-based frustules. Polyamines in diatoms appear to be species-specific, which play an important role in the formation of frustules with species-specific patterns [21]. In other words, biosilicification in diatoms might be modulated by the specific structure of polyamines involved in the precipitation process [27].

Sponge spicules also possess highly hierarchical and organized siliceous nanostructures. The laminated spicule structure consists of alternating layers of silica and organic material [28]. Although the mechanism of biosilicification in sponges is distinct from that of silica formation in diatoms [29], organic amines have also been identified from the marine sponge *Axinyssa aculeata* [30]. These polyamines separated from sponge can deposit silica and the polyamine-derived macromolecules are chemical factors involved in silica deposition in sponges [30].

Phospholipids also play an important role in biosilicification [31]. Diatom silicification takes place in the silica deposition vesicle [32], whose membrane, called the silicalemma, consists of a typical lipid bilayer [33]. The overall outline of diatom's silica structure is determined by shaping of this kind of membrane-bound compartment [34]. Hildebrand et al. found that the silicalemma is tightly clung to siliceous structures in areas where silica is deposited [35]. This indicates that membrane components of silica deposition vesicle could become part of the silica structure [36], [37]. Recently, X-ray photoelectron spectroscopy (XPS) [38] and solid state NMR (SSNMR) [39] studies were performed on diatom cells for analyzing the chemical composition of the diatom surface. The XPS analysis revealed a high concentration of lipids present as a structural part of the cell wall in the form of carboxylic esters. The SSNMR study also demonstrated that lipids are tightly associated with silica, even after harsh chemical treatment. All these imply that phospholipids may involve in the amines-mediated biosilica deposition in diatoms. [40], [41].

Although the phase separation model successfully explain the important aspects of silica patterning in diatoms, biosilica in diatoms and sponges have other nanometer-scale details, and their nuanced structural and biological functions are well beyond the current ranges used in advanced materials [42]. Taking the centric diatom *Thalassiosira eccentrica* as an example, the ground-plan of its areolae is a two-dimensional system of hexagonal meshes [43]. Moreover, starting from this ground plan, the vertical growth of areolae walls and the horizontal extension on the distal side of areolae walls occur in sequence. It indicates that the asymmetrical development of silica deposition can be well achieved in diatom silicification [44]. However, it is still difficult to understand how the differentiation of solid siliceous structures would occur in different directions [34].

In this study, dodecylamine (DA) and phospholipid (PL) were selected as model organic additives to initiate phase separation and influence silica precipitation. Phospholipid, which has a hydrophilic head and two hydrophobic tails, is a major component of all the plasma membranes including the silicalemma in diatoms and sponges [45]. The goal of this study is to examine the effect of phase separation of biosilicification-associated model organic components on the development of silica morphology, and thus to reveal the contribution of the organic phase separation to growth differentiation of biogenic silica. As a consequence, asymmetrical discus-like silica particles with controlled aspect ratios were indeed obtained during the phase separation of PL and DA, and the morphological evolution of the deposited silica from spherical through sunflower-looking to discus-like features were also exhibited at different conditions. Since the organic amines, membrane lipids, and the phase separation process are the important features of diatom silicification, our results may be useful for a deeper insight into biosilicification.

## Materials and Methods

### Materials

All starting chemicals were purchased from Sinopharm Chemical Reagent Co., Ltd, and used as received without further purification. Phospholipids (PL) are of biotech grade while all other reagents, such as ethanol, dodecylamine and tetraethyl orthosilicate, are of analytical grade. Deionized water was also used in these syntheses. For all experiments, glassware was cleaned with aqua regia (3∶1 HCl/HNO_3_), rinsed thoroughly with ultrapure water, and oven-dried overnight before use.

### Preparation

In a typical biomimetic synthesis, 0.10 g of PL and 0.16 g of DA (0.863 mmol) were dissolved in 30 mL of ethanol through ultrasonification, and then stirring for about 5 min in a closed 100 mL flask until the solution became clear (Fig. S1a in Supplementary Information). Afterwards 30 µL of TEOS (0.134 mmol, 2.2 mM) was injected into the solution using a 50 µL syringe with stirring. In succession, 30 mL of H_2_O was added to the above solution to obtain a turbid suspension (Fig. S1b). This suspension was then heated in a 80°C thermostated water bath, and became clear again with the increase of temperature (Fig. S1c). After 24 h of thermostated reaction, the solution was moved out of the water bath, and cooled down to room temperature naturally. As the temperature of the solution lowered, a white turbidness gradually appeared. Notably, the turbidness could be explicitly distinguished after the flask was cooled down for an hour at room temperature (Fig. S1d). Nevertheless, the centrifugated precipitate could dissolve in ethanol, and thus no silica could be obtained in this case, indicating that the isolated precipitate should be organics, i.e., an undissolvable organic phase was first formed at room temperature. After the flask was continuously stationed for another 1 day (Fig. S1e), the resultant particles were isolated by centrifugation, cleaned by three cycles of centrifugation/washing/redispersion in ethanol, and dried at room temperature for 1 day in vacuum. The obtained sample was named as sample L5. For other morphogenesis of silica structures, the same synthetic procedures were deployed except that some experimental parameters were varied. The detailed experimental conditions and the corresponding aspect ratios of the silica particles are listed in Table S1. Moreover, in order to understand the detailed microstructures, some samples were also calcined at 550°C in air for 6 h to remove the occluded organic components, and XRD and nitrogen physisorption analyses were performed.

### Characterization

Several analytical techniques were used to characterize the products. Field emission scanning electron microscopy (FESEM) (JEOL JSM-6700 F) was applied to investigate the size and morphology. Transmission electron microscope (TEM) images were obtained on a JEM 2010 transmission electron microscope with an accelerating voltage of 200 kV. The samples for the TEM measurements were prepared by dropping a few drops of sample suspension with ethanol as the solvent on a copper grid, and the solvent was allowed to evaporate to dry state before analysis. The powder X-ray diffraction (XRD) patterns of the samples were recorded with a Japan Rigaku TTR-III X-ray diffractometer 0.154056 nm), employing a scanning rate of 0.02°s^−1^ in the 2θ range 0.8–10°. Infrared spectra were collected using a Nicolet 8700 FT-IR spectrometer on KBr pellets. Thermogravimetric and differential thermal analysis (TG-DTA) was carried out using a SDTQ 600 TG/DTA thermal analyzer (TA, USA) with a heating rate of 10°C/min from room temperature to 800°C in a flow air atmosphere. N_2_-sorption isotherms of the samples were measured by using a Micromeritics Tristar II 3020 M instrument at liquid-nitrogen temperature. From the adsorption isotherm, the Barrett-Joyner-Halenda theory (BJH) was used to calculate the mesopore volume and its size distribution. Specific surface areas were calculated by using the Brunauer-Emmett-Teller (BET) method in the relative pressure range of P/P_0_ = 0.05–0.3. Pore volumes were obtained from the volumes of N_2_ adsorbed at P/P_0_ = 0.95 or in the vicinity. The dispersibility of suspensions was estimated by dynamic light scattering (DLS, DYNAPRO-99).

## Results and Discussion

**Figure 1:**
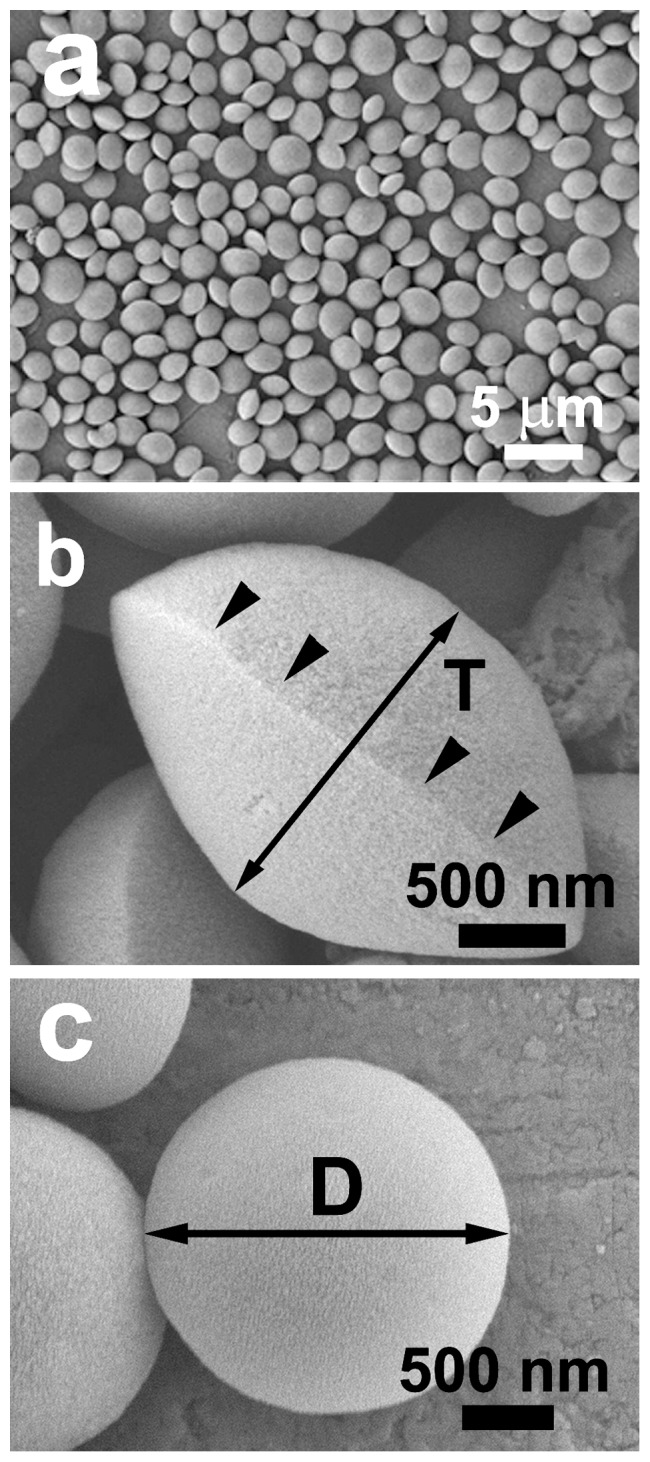
SEM images of discus-like silica particles (sample L5): low magnification (a), the side- (b) and front-view (c) observations of individual particles.

**Figure 2:**
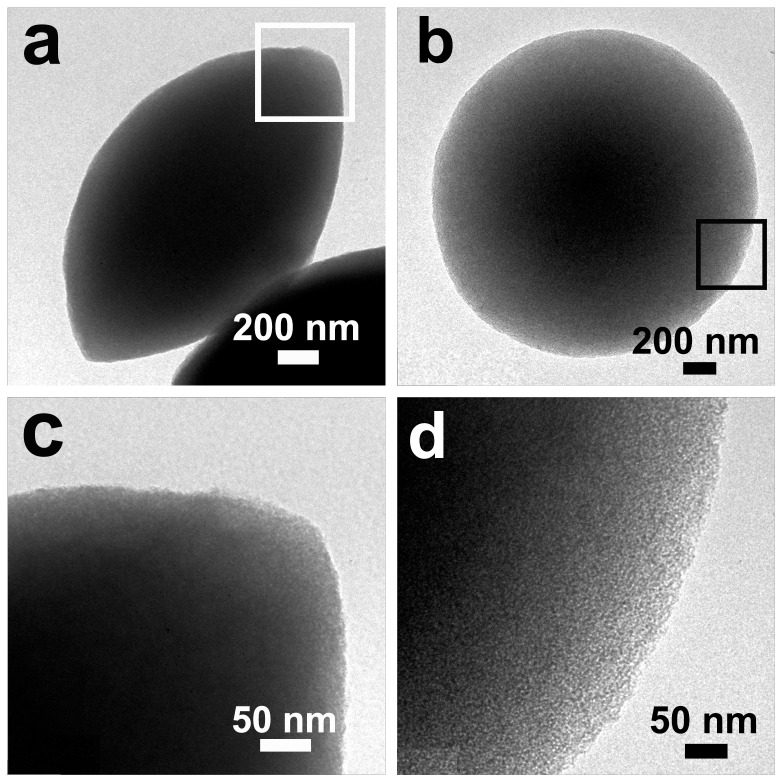
TEM images of individual particles in sample L5 by a side (a) and front (b) view, and their local high-magnification images (c, d).

**Figure 3:**
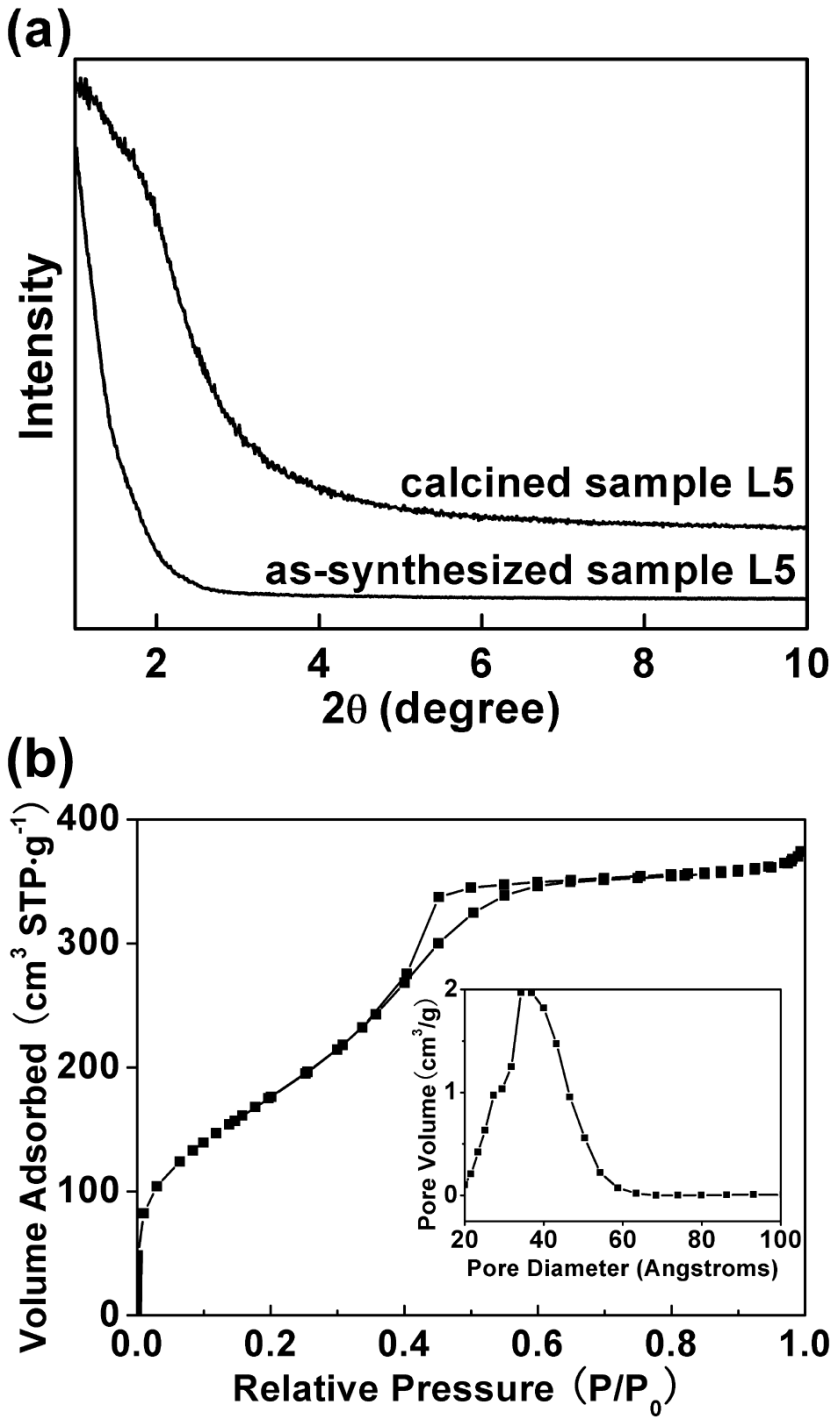
XRD patterns (a) of sample L5 before and after calcined and N_2_ sorption isotherms (b) of the calcined sample L5.

Figure 1a depicts the low-magnification FESEM image of sample L5. The product is solely composed of the discus-like particles with a diameter of 2.0–3.0 µm, and no aggregation among the particles occurs. Figure 1b and c present the side and front view of an individual particle, respectively. The discus-like morphology is further confirmed and a ridge between the two halves is visible (indicated by black arrowheads in Fig. 1b). The ratio (D/T, *i.e*. aspect ratio) of particle diameter ("D" in Fig. 1c) to thickness ("T" in Fig. 1b) is 1.60±0.06. It should be pointed out that the two halves are not completely symmetric (e.g., Fig. 1b), which is also observed in the corresponding TEM image (Fig. 2a). The TEM analyses (e.g., Fig. 2b) also show that the discus-like particles are not hollow, but solid. Fig. 2c and d depicts the local-magnification TEM images of the areas framed in Fig. 2a and b, respectively. The disordered pores are obviously discernable, and no resolved diffraction peaks can be observed in the XRD patterns including calcined sample L5 (Fig. 3a), indicating that the arrangement of the pore channels may be random [46]. Fig. 3b presents the N_2_ adsorption–desorption isotherm with the inset of the BJH pore size distribution of the calcined sample L5. One can see a typical type IV isotherm with a N_2_ hysteresis loop in the calcined sample, indicating the mesoporous property [47]. The adsorption isotherm shows a well-defined capillary condensation step at relative pressure (P/P_0_) of 0.40–0.50, corresponding to the pore size of 3.3 nm. The Brunauer-Emmett-Teller (BET) surface area is calculated at 730 m^2^·g^−1^ and the pore volume is 0.62 cm^3^·g^−1^. Therefore, the silicified product is an asymmetrical discus-like structure possessing disordered mesoporous character.

**Figure 4:**
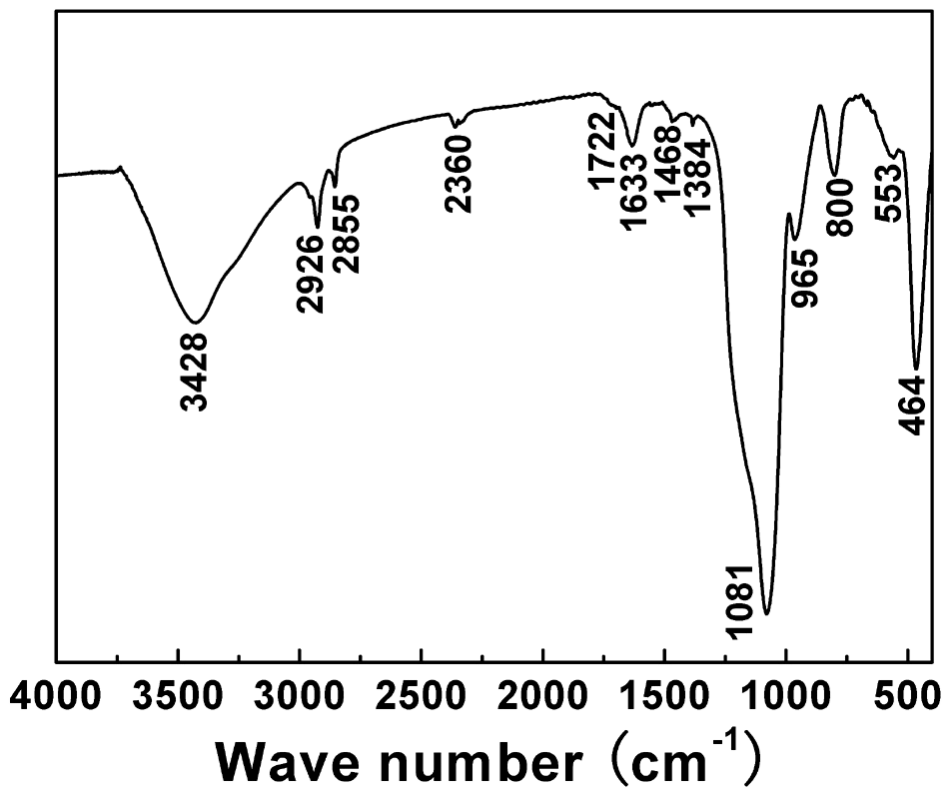
FTIR spectrum of the discus-like particles (sample L5).

The FT-IR spectrum (Fig. 4) of sample L5 displays three characteristic peaks of silica: Si-O-Si asymmetric stretching at 1081 cm^−1^, symmetric stretching at 800 cm^−1^, and Si-OH stretching at 965 cm^−1^ [48]–[51]. The H-bonded OH groups with various OH···H distances are responsible for the intense absorption at 3428 cm^−1^, and the band at 1633 cm^−1^ is due to the δ(HOH) of physisorbed water [52]. Bands detected at 2926 and 2855 cm^−1^ are assigned to the stretching vibrations of the CH groups, which indicate the existence of organic components [50]. The characteristic vibration of C-C bonds at 1468 cm^−1^ is also observed. Moreover, the bands at 553 and 1722 cm^−1^ can be assigned to the O-P-O bond and the carbonyl group, respectively, both of which should originate from phospholipid molecules [53].

**Figure 5:**
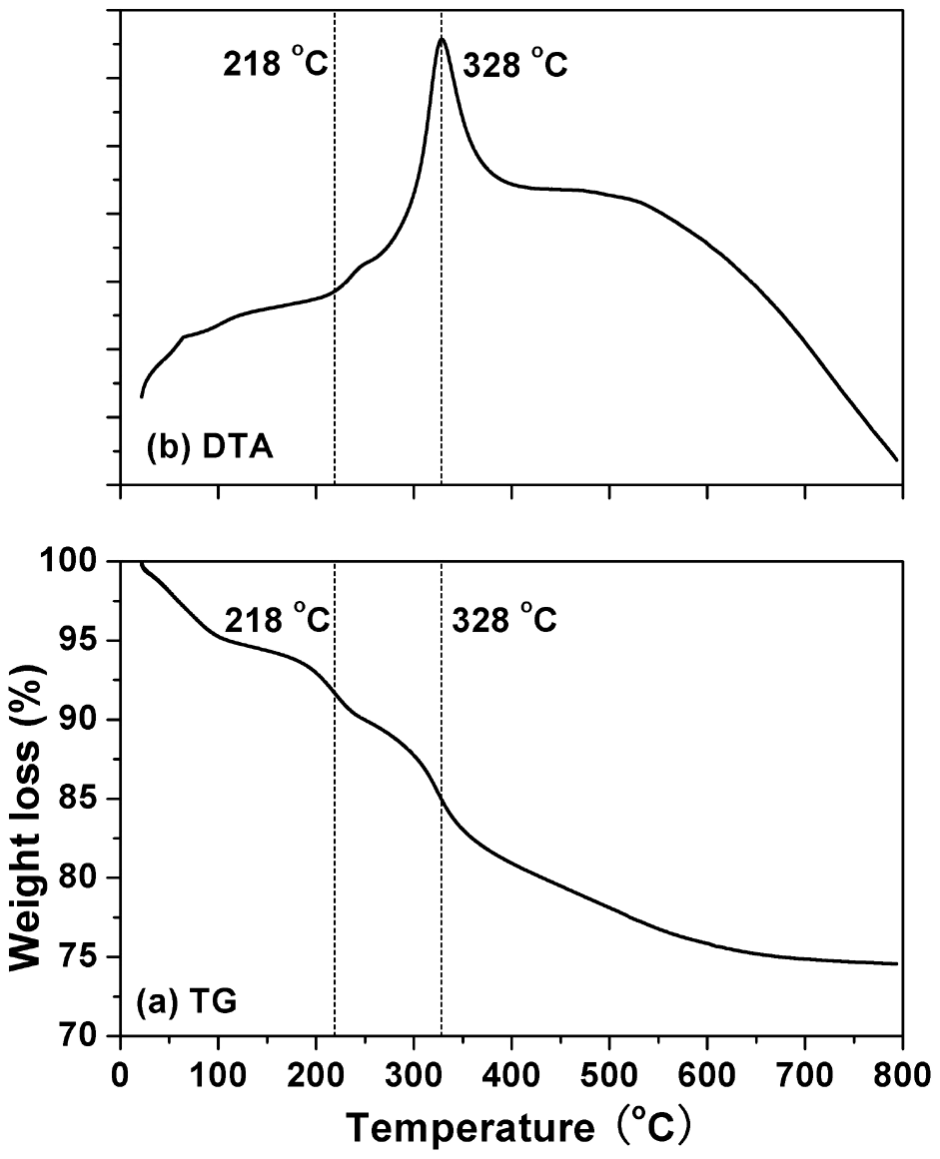
TG (a) and DTA (b) curves of sample L5.

Figure 5 presents the TG-DTA curves of the original silica particles (sample L5). The TG curve reveals ∼25.4% total weight loss from room temperature to 800°C. A ∼5.2% of weight loss from room temperature to 120°C and the corresponding endothermal peak at 50°C in DTA curve indicate the evaporation of the surface-adsorbed water and ethanol. The small endothermic peak at 218°C in the DTA curve is believed to originate from the organic component decomposition and/or the polycondensation of the silica network [54]. The exothermic peak at 328°C can be ascribed to the combustion of the incorporated organic substances [55]. The weight loss at temperatures above 400°C (6.3%) is generally due to the further condensation and dehydration of silanol groups [56]. FT-IR and TG-DTA analyses of the as-synthesized product confirm the co-existence of silica and organic components, indicating the formation of an organic-inorganic composite.

**Figure 6:**
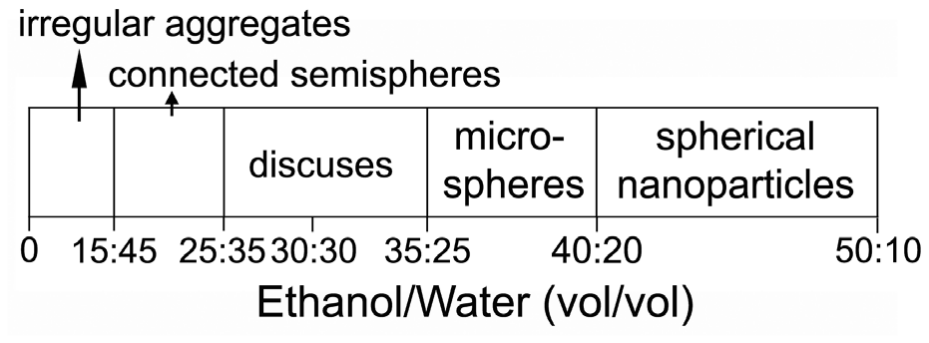
Schematic illustration of the product morphology dependence on ethanol/water volume ratio. The amounts of PL, DA, and TEOS, and the total solution volume were fixed at 0.10 g, 0.16 g, 30 µL, and 60 mL, respectively.

**Figure 7:**
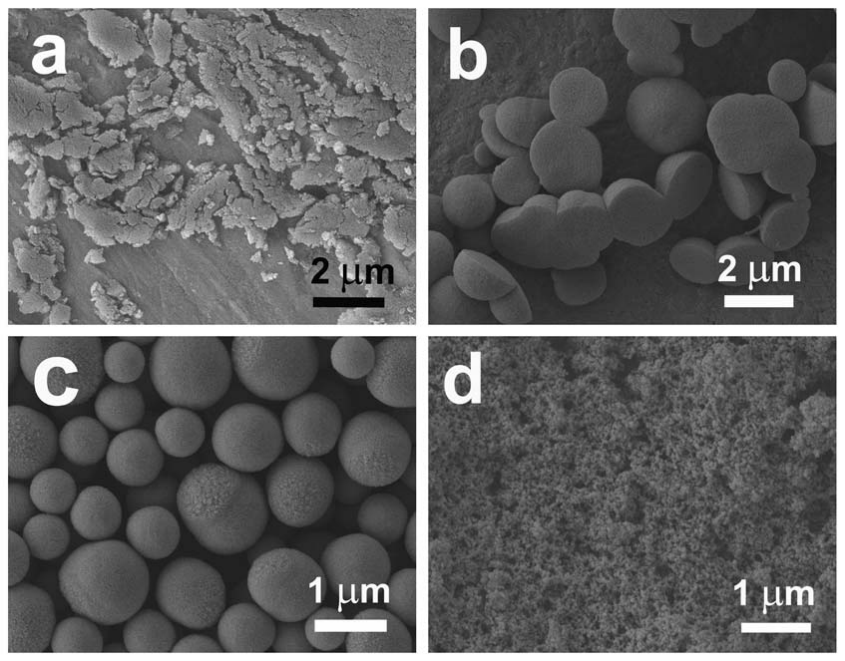
SEM images of the samples prepared at different volume ratios of ethanol to water: (a) 15/45; (b) 25/35; (c) 35/25 and (d) 40/20.

Moreover, our results also show that the asymmetrical structured silica including semispherical or discus-like particles can be obtained in a relatively broad range of ethanol/water volume ratio (E/W), as shown in Figs. 6. When the E/W is varied between 15/45 and 25/35, the interconnected semispherical particles are always obtained (Fig. 6 and 7b). This is due to the fact that the lower the E/W ratio, the more the precipitated turbidness. As a result, more and more oil droplets are formed. Therefore, the silicified particles are inclined to connect each other. When the E/W is under 15/45, however, both PL and DA can not be well dissolved, and an irregular aggregate is formed (Fig. 6 and Fig. 7a). Conversely, when the E/W is over 35/25, the morphologies of the products change from discus (Fig. 1) to microsphere (Fig. 6 and Fig. 7c). This is possible because higher ethanol concentrations facilitate the dissolution of organic components, and do not favor the formation of the oil-water interface [57], [58]. Further increasing E/W to 40/20 leads to the formation of ca. 60-nm-diameter spherical nanoparticles (Fig. 6 and Fig. 7d), which is probably because of the shrinking effect of ethanol at such a high E/W [59], [60].

**Figure 8:**
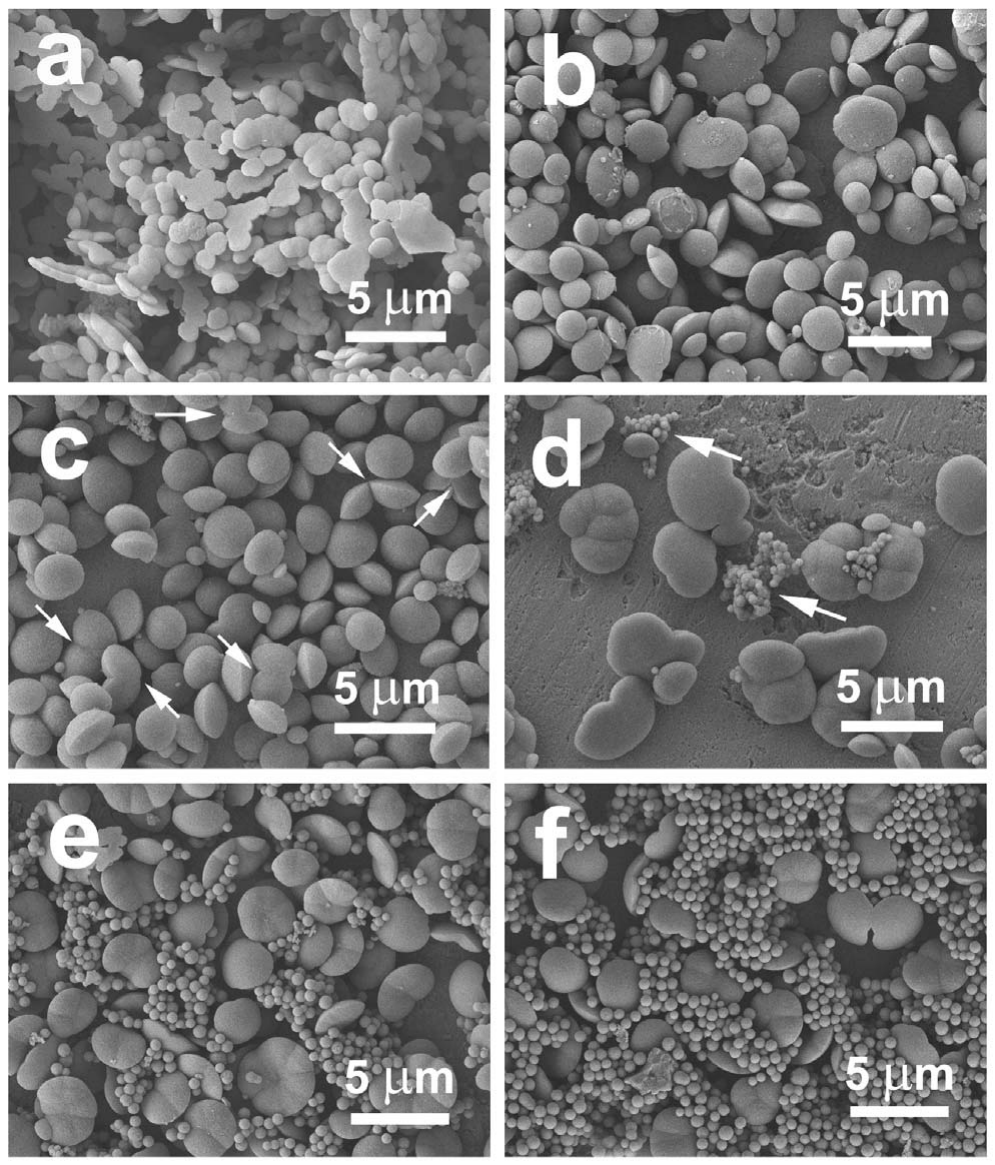
SEM images of the samples prepared with different concentrations of TEOS: (a) 0.7 mM; (b) 1.5 mM; (c) 3.0 mM; (d) 3.7 mM; (e) 4.5 mM and (f) 5.2 mM.

The concentration of silica precursor (TEOS) is also another important factor for the morphogenesis of the asymmetrical silica (Fig. 8). Monodisperse discus-like particles can be obtained with a TEOS concentration of 2.2 mM (sample L5, Fig. 1). However, the interconnection among the particles becomes more significant in the case of both higher and lower concentrations of TEOS. With the decrease of TEOS concentration to 1.5 mM, the development of the two halves is insufficient and the deposition region of silica is predominantly confined to the water/organics interfaces. Therefore, the obtained particles become thinner (as indicated in Fig. 8b) and the siliceous extension at the interfaces makes some particles interconnect each other. Further interconnection among the silica wafers occurs with decreasing the TEOS concentration to 0.7 mM (Fig. 8a). Nevertheless, it is almost impossible to collect any precipitate as the concentration is lower than 0.7 mM. Conversely, as the concentration of TEOS is over 2.2 mM, the particles show better growth with an increase in thickness and diameter (Fig. 8c-f): while the TEOS concentration is 3.0 mM, particles with obvious ridges exhibit asymmetrical discus-like shapes, and some asymmetrical particles fuse together along their ridges (as indicated by arrows in Fig. 8c). Further increasing the concentration to 3.7 mM results in the extra formation of spherical particles together with the asymmetrical aggregates of silica (as indicated by arrows in Fig. 8d). More spherical particles with lager diameters can be observed as 4.5 mM or 5.2 mM of TEOS is used (Fig. 8e and f). The emergence of extra spherical silica at the higher concentrations of TEOS can be ascribed to the independent nucleation and growth of silica in the reaction solutions. We have noted that the reaction solution with 3.7, 4.5 or 5.2 mM of TEOS cannot become clear under the same heating conditions. In other words, silica precipitation has occurred before the cooling-down, which may result in the formation of the extra silica spheres at the higher TEOS concentrations. In summary, the morphology of silica is sensitive to the concentrations of TEOS over the range of 0.7 to 5.2 mM. Thicker and more robust siliceous structures are formed with increasing the concentrations of silica precursor. Similar phenomenon has been found by Finkel et al [61] when they tried to quantify silicification in marine diatoms. The frustules became more heavily silicified with increasing silicate concentrations over the range of 0.02–1.1 mM. Therefore, changes in the frustules thickness of diatoms may provide a paleoproxy for surface silicate concentrations under conditions where they lived [61].

**Figure 9:**
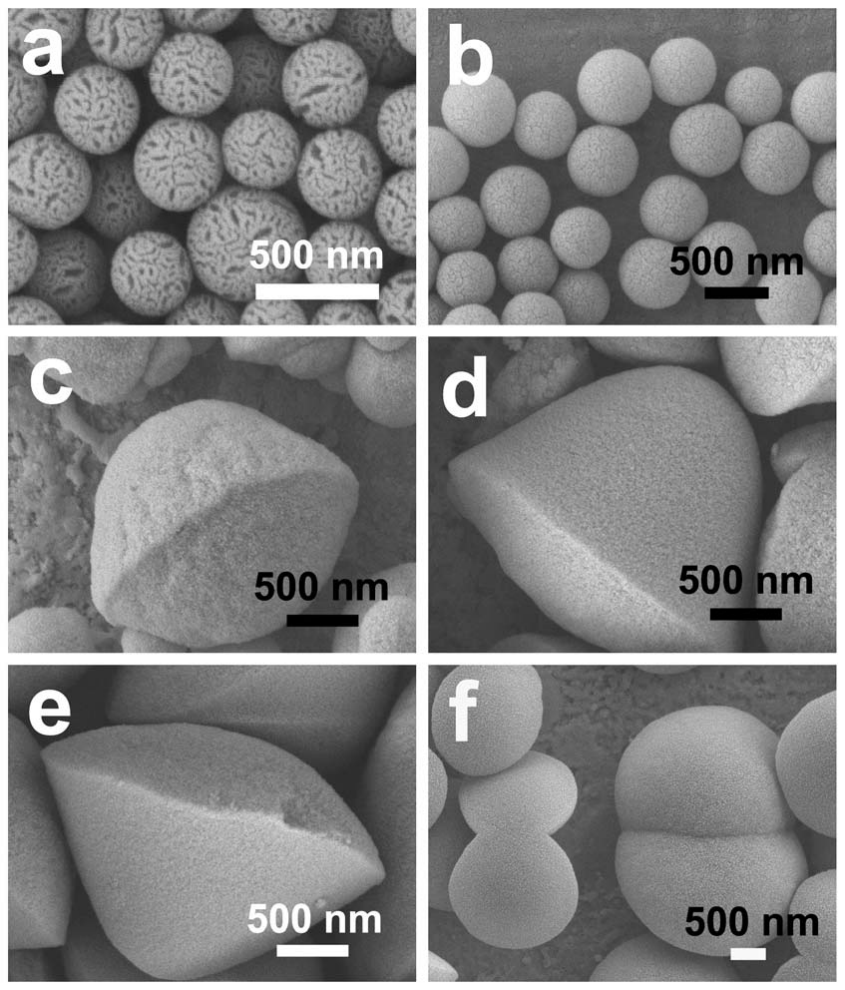
SEM images of the samples obtained at different concentrations of PL: (a) 0.00 g/L; (b) 0.35 g/L; (c) 0.70 g/L; (d) 1.00 g/L; (e) 1.35 g/L; (f) 2.00 g/L.

**Figure 10:**
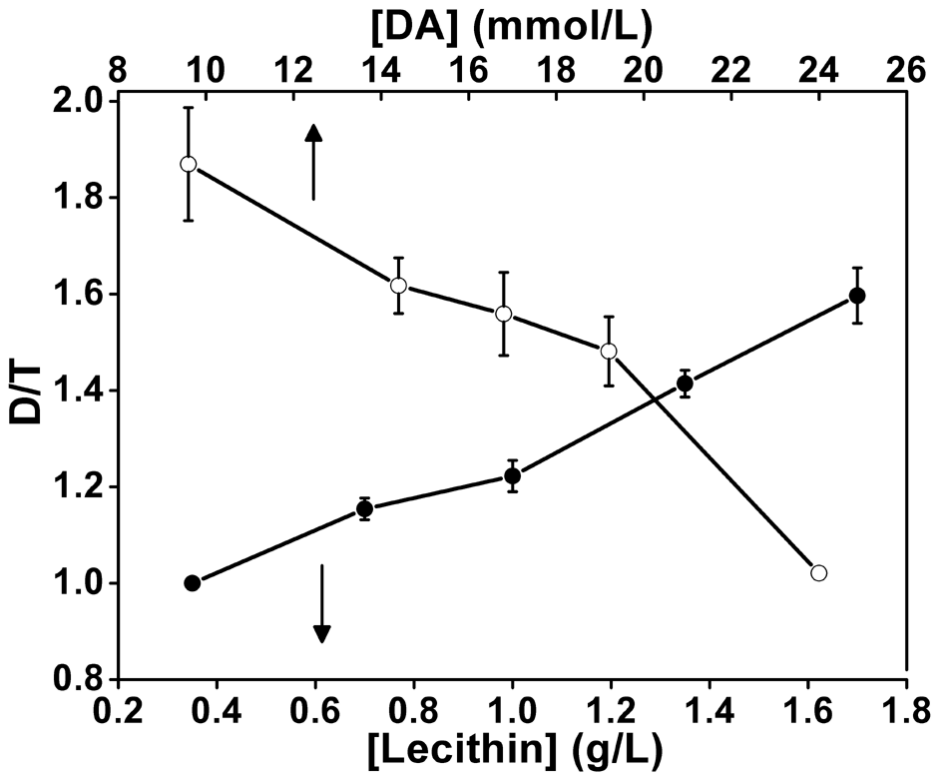
Relationship between the aspect ratio of particles and the concentration of PL or DA in the mixed solvent of 30 mL ethanol and 30 mL water. The amount of DA was fixed at 0.16 g for the solid circular symbols. The amount of PL was fixed at 0.10 g for the hollow circular symbols. The aspect ratio of particles was the average value obtained in the SEM images, and at least 50 particles were measured in each case.

For a better understanding of the morphogenesis details of the asymmetrical siliceous structures, a series of experiments with different concentrations of PL or DA were carried out. The experimental details are depicted in Table S1. Increasing PL concentration from 0 to 1.70 g/L (samples L1-L5; Fig. 9a-e and Fig. 1) leads to an increase in the aspect ratio of the obtained particles (see the line symbolized with ‘•’ in Fig. 10). Many connected particles appear with the further increase of PL concentration to 2.0 g/L (sample L6; Fig. 9f), so their aspect ratios are not calculated. It should also be pointed out that the asymmetry between the two halves is much more significant in Fig. 9c-e relative to Fig. 1. Nevertheless, cracked spheres are prepared without the addition of PL (Fig. 9a), which can be determined by the shape of the initial DA micelle [62]. On the other hand, when the concentration of PL and the pH of initial reaction mixture are fixed at 1.70 g/L and 11.6, respectively, the particles become thinner with decreasing DA concentration (see the line symbolized with ‘○’ in Fig. 10). In the absence of DA, silica films are finally produced (data not shown). It is probably due to the fact that the property of organic aggregates and the DA concentration in the system pose an important influence on the silica morphogenesis. DA can interact with PL in solution [63], [64]. The incorporation of DA molecules can introduce more amino groups into the organic aggregates. These amino groups further interact with silanol groups of silicates, and induce the preferential deposition of silica at the organic interface. Meanwhile, increasing DA concentration inevitably leads to more DA molecules coprecipitation into silica and/or anchoring to the surfaces of siliceous structures. These also favor the growth and thickening of the siliceous structures. As a result, the thicker silica structures can be formed at the higher concentration of DA. In contrast, thinner silica particles with higher diameter/thickness (D/T) ratios are obtained at the lower concentration of DA. Meanwhile, the excessive extension of silica at the interface causes the connection of the neighboring particles to be easier. Especially, in the absence of DA, silica deposition predominately occurs at the PL interface owe to the electrostatical interaction between Si-O^−^ groups from silicates and ammonium head groups from PL molecules [65]. Silica deposition is confined to the extension of silica at the interface. The connection of the neighboring particles occurs commonly and the film-like siliceous structures are finally obtained without DA. On the basis of the above results, it can be concluded that both PL and DA are indispensable factors during the formation of asymmetrical silica particles. Moreover, it can be seen from Fig. 10 that these two additives display opposite effects on the aspect ratio of the resultant particles. The change of aspect ratio can be considered as an indication of silica asymmetrical growth in different directions. Therefore, the asymmetrical growth of siliceous structures can be well controlled by changing the proportion of organic components PL and DA in our experiments. In the past few years, the fabrications of asymmetrically structured silica have been reported. Non-spherical silica Janus colloids, for instance, were produced by asymmetric wet-etching at the wax/water interface [66]. However, it is not achieved directly by the asymmetrical deposition of silica. Wang et al. [67] used a single-step emulsion templating method creating budded mesoporous silica capsules with the protruding stumps formed in particular orientations, and the radiolaria-like morphology of silica with multicellular structured spines has also been obtained [68]. However, to the best of our knowledge, no report on the preparation of asymmetrical silica structures in the presence of phospholipid and organic amine can be found, and the aspect ratio (diameter-to-thickness ratio) of the obtained particles can be finely controlled by tuning the feeding amount of organic components (Fig. 9 and 10).

In our biomimetic experiments, PL and DA are used as the biosilicification-associated model organic components to form PL-DA composite emulsion by a deliberate heating-cooling process (see the experiment details and Fig. S1) and create oil-water interface at room temperature for the deposition of silica. Specifically, PL can dissolve in the ethanol/water mixture at 80°C [69]. Therefore, a 80°C pretreatment temperature was selected to promote PL dissolution and reinforce PL-DA interaction. In fact, the solution became clear during continuous heating process, which suggests that neither organic turbidness nor silica precipitation formed in this process (Fig. S1c). After the flask is removed out of the water bath and cooled down naturally, however, white organic turbidness appears with the gradual decrease in temperature, and the phase separation can be directly observed at room temperature (Fig. S1d) [57]. Furthermore, our DLS results also reveal that the larger micelles (1781.5±712.4 nm in diameter) indeed occur in the suspension at room temperature, confirming the phase separation process present. It has been well known that the dodecyl chains of DA molecules can interact with the PL hydrophobic chains by van der waals force, while their NH_2_ or NH_3_^+^ heads interact with P-O^−^ groups of PL by hydrogen bonding and electrostatic interaction [63]. Therefore, in such physico-chemical environment, the organic emulsion is formed, and subsequently the hydrolysis of TEOS occurs near the oil/water interfacial region owe to the electrostatical interaction of Si-O^−^ from silicates and the ammonium groups from PL and DA molecules [65]. As previously reported, asymmetrical polystyrene particles with flattened shapes were produced at an oil-water interface [70]. Driven by surface tension [57], [70], the particles appear to be spreading at the fluid interface, which leads to the appearance of ridge and subsequent formation of discus-like particles. It should be pointed out that although the preheating process was carried out first, the formation of organic turbidness and silica precipitation did occur at room temperature. These results suggest that the precipitation of asymmetrical silica structures can be achieved by phase separation of the organic components (e.g., Fig. 1, Fig. 9c-f and Fig. 10). It appears that the interaction between the different organic molecules and their phase separation can significantly affect physico-chemical growth environment of the siliceous structures, and finally control the silica morphologies [71].

**Figure 11:**
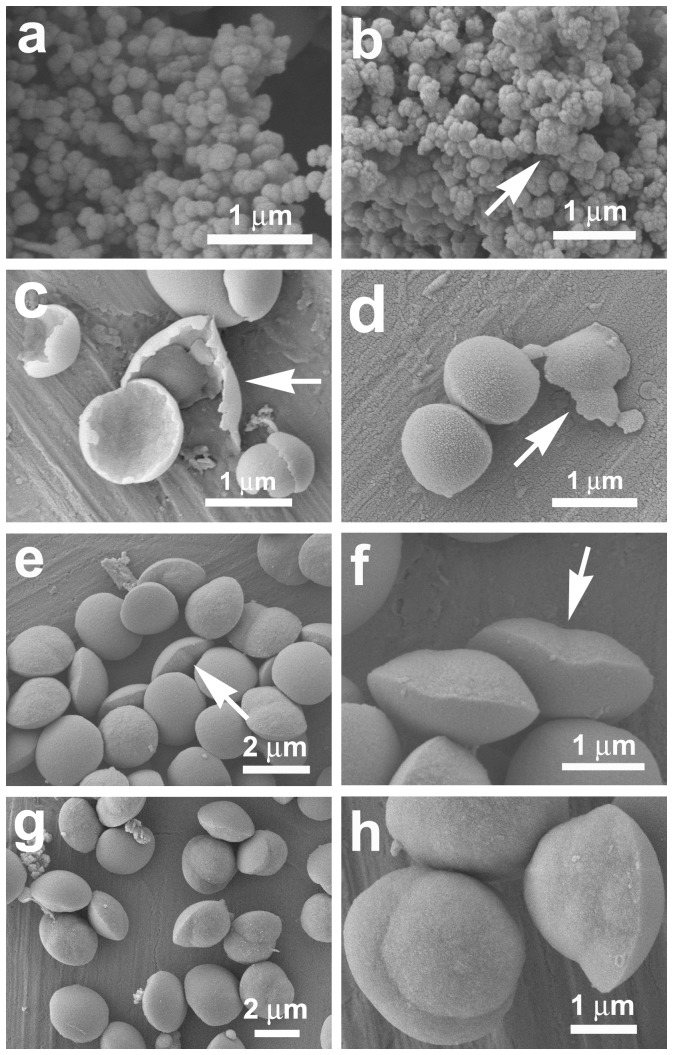
FESEM images of silica particles after the reaction mixtures were first heated at 80°C for 1 day and then cooled down at room temperature for (a,b) 1.25 h; (c) 1.5 h; (d) 1.75 h; (e, f) 2 h and (g, h) 2.25 h.

To further understand the formation details of the discus-like silica particles, some time-dependent silicification experiments are also carried out. It is found that the reaction solution turns gradually turbid during the cooling process at room temperature, and the precipitate obtained by centrifugation after 1 h of standing is organic components because the precipitate can completely dissolve in ethanol. However, the precipitate obtained after 1.25 h of standing can incompletely dissolve in ethanol, indicating that the silicified structures have formed. SEM analysis reveals that the silicified structures consist of small silica particles of ca. 250 nm in diameter (Fig. 11a), and the interconnection of the particles leads to the appearance of some larger aggregates with diameter above 400 nm, as arrowed in Fig. 11b. After 1.5 h of reaction, however, some particles with thin margin can be found (arrowed in Fig. 11c), indicating that the morphological development of the particles may occur at the oil/water interface. When the silicification system continues standing for 1.75 h, the particles have developed into discus-like embryos with diameter up to 1 µm (Fig. 11d). Moreover, a few discus-like particles with the expanding margin can be clearly observed (typically arrowed in Fig. 11d), further supporting that the formation of the discus-like structures occurs at the oil/water interface. Further prolonging the standing time to 2 or 2.25 h leads to the appearance of the well-developed asymmetric discuses of ca. 2 µm in diameter, and many of them exhibit conjoined structures (Fig. 11e-h). Combined with the results depicted in Fig.1, it is not difficult to find that at the oil/water interface, aggregation, fusion and margin expansion of the small siliceous particles, as well as further growth lead to the monodisperse perfect discus-like asymmetric structures.

**Figure 12:**
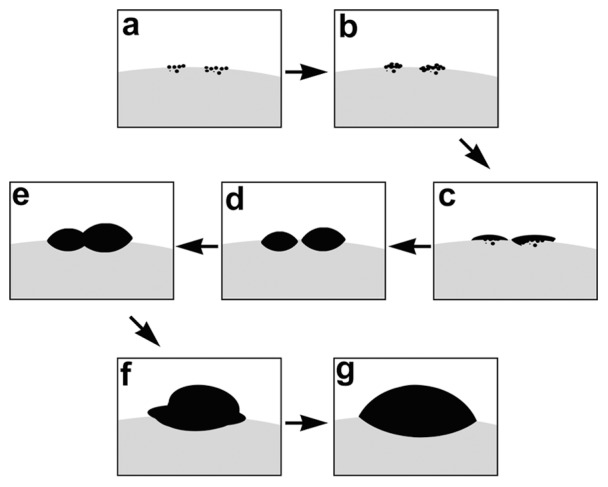
Schematic illustration of the formation of discus-like silica particles. The organic precipitates, silica particles and reaction solution are stained in gray, black and white, respectively.

On the basis of our time-dependent experiments, a tentative mechanism is proposed and illustrated in Fig. 12 for the formation of discus-like asymmetric silica. Namely, when the bulk solution is cooled down naturally, the hydrolysis of TEOS and the precipitation of silica occur slowly near the oil/water interfacial region with the phase separation of organic components. The silica formation begins with the appearance of small particles (Fig. 12a, Fig. 11a). With the growth and aggregation of them, larger aggregates of silica particles can be formed (Fig. 12b, Fig. 11b). Further growth of these aggregates get their surfaces smoother, and the growth environment (oil/water interfacial region) facilitates their expansion at the oil/water interfaces. Therefore, flake-like silica structures appear (Fig. 12c, Fig. 11c), and further develop into discus-like particles with a diameter of ca. 1 µm, which is much smaller than the final product (2–3 µm) (Fig. 12d, Fig. 11d). As the margin expansion process continues, several neighboring particles (e.g., two particles) are joined together to form the “conjoined structures” (Fig. 12e, Fig. 11e-f). The further fusion and growth of the conjoined structures lead to discus-like particles with diameter above 2 µm (Fig. 12f, Fig. 11g-h). Finally, the fully development of their two halves results in the formation of well-defined asymmetric discus-like structures of 2–3 µm in diameter (Fig. 12g, Fig. 1).

### Implication for biosilicification

Silicification in diatoms is a complicated process involving architecture design from nano- to microsize level [72]. The siliceous structures formed in different scales and stages can be unified in the mineralization system of diatoms, and finally assemble into hierarchical and multifunctional frustules. The valve development of *Thalassiosira eccentrica* can be divided into three stages. Formation of base layer (areolae) defines the structure in the x, y plane (Stage 1), and subsequent deposition (Stage 2) involves expansion in the z axis but only in one direction [34], [43], [44]. During the development of the outer layer (Stage 3), however, the differentiation of the plane occurs again, forming a right angle to the previous plane (Stage 2) and lying parallel to the base layer (Stage 1) [43]. The formation of the two-dimensional system of hexagonal meshes (areolae) in stage 1 can be well explained by the phase separation model [25]. However, it is not clear whether this model is also suitable for the asymmetrical precipitation of silica including vertical expansion in stage 2 and horizontal growth in stage 3.

Space-limited by the membrane-bound compartment and promoted by organic amines, siliceous base layer with pores in a hexagonal arrangement formed during the phase separation of organic droplets [40], [41]. However, the role of organic amines and phospholipids on biosilicification may be not restricted to influencing the development of base layer. Our experiments exhibit the controlled deposition of asymmetrical silica particles during the phase separation. The asymmetrical particles emerge as the concentration of PL is over 0.70 g/L. The addition of PL favors the morphology transition from spherical to discus-like particles and the aspect ratio regularly increases with increasing the concentration of PL (e.g., Fig. 9). These results show that phospholipids can provide distinct chemical influences in organic-amine-induced silica precipitation [34], [43], [64]. That is, their aspect ratios can be easily adjusted through varying the stoichiometric compositions of the mineralization system (including DA and PL, Fig. 9 and 10). And the degree of fusion among the neighboring siliceous structures is drastically affected by the concentration of silica precursor (Fig. 8). Therefore, it can be presumed that the phase separation of organic droplets is still an important process for the oriented differentiation of silica. In other words, the phase separation model may be broadened to explain the formation of siliceous structures in the last two stages.

## Conclusions

A series of experiments were accomplished by introducing PL and DA into the reaction system to initiate phase separation of organic components and influence the morphogenesis of silica. The results show that this phase separation process leads to the formation of asymmetrically non-spherical silica structures, and the aspect ratios of the asymmetrical structures can be well controlled by varying the concentrations of PL and DA. A tentative mechanism is also proposed based on the time-dependent experiments. Moreover, controlling the degree of fusion among the neighboring siliceous structures can be achieved via modulating the concentration of silica precursor (TEOS) in the silicified region. Based on the special importance of phospholipids (e.g., silicalemma), organic-amines and the phase separation process for biosilicification, our results suggest that in addition to explaining the biosilica nanopatterning, the phase separation process may be also involved in the growth differentiation of siliceous structures in specific directions. This provides a new insight into the mechanism of biosilicification.

## Supporting Information

Figure S1 **Digital pictures: (a) the clear solution after DA, TEOS and lecithin were dissolved in 30 mL of ethanol; (b) the turbid suspension obtained after a 30 mL of H_2_O was added into the ethanol solution; (c) the turbid suspension became clear by heating treatment in 80°C water bath for 24 hours; (d) the turbidness appeared again after the clear solution was cooled down at room temperature for 1 h, and the temperature of the suspension is close to room temperature; (e) much more turbidness was obtained after a 24 h of cooling.**
(DOC)

Table S1 **Experimental conditions and corresponding aspect ratios of the silica particles (Ethonal/water = 30∶30 vol/vol).**
(DOC)
