# Supplementary figures and images for: Formation of Asymmetrical Structured Silica Controlled by a Phase Separation Process and Implication for Biosilicification
Source: PLoS One. 2013 Apr 9;8(4):e61164. doi: 10.1371/journal.pone.0061164 (PMC3621999; doi:10.1371/journal.pone.0061164)

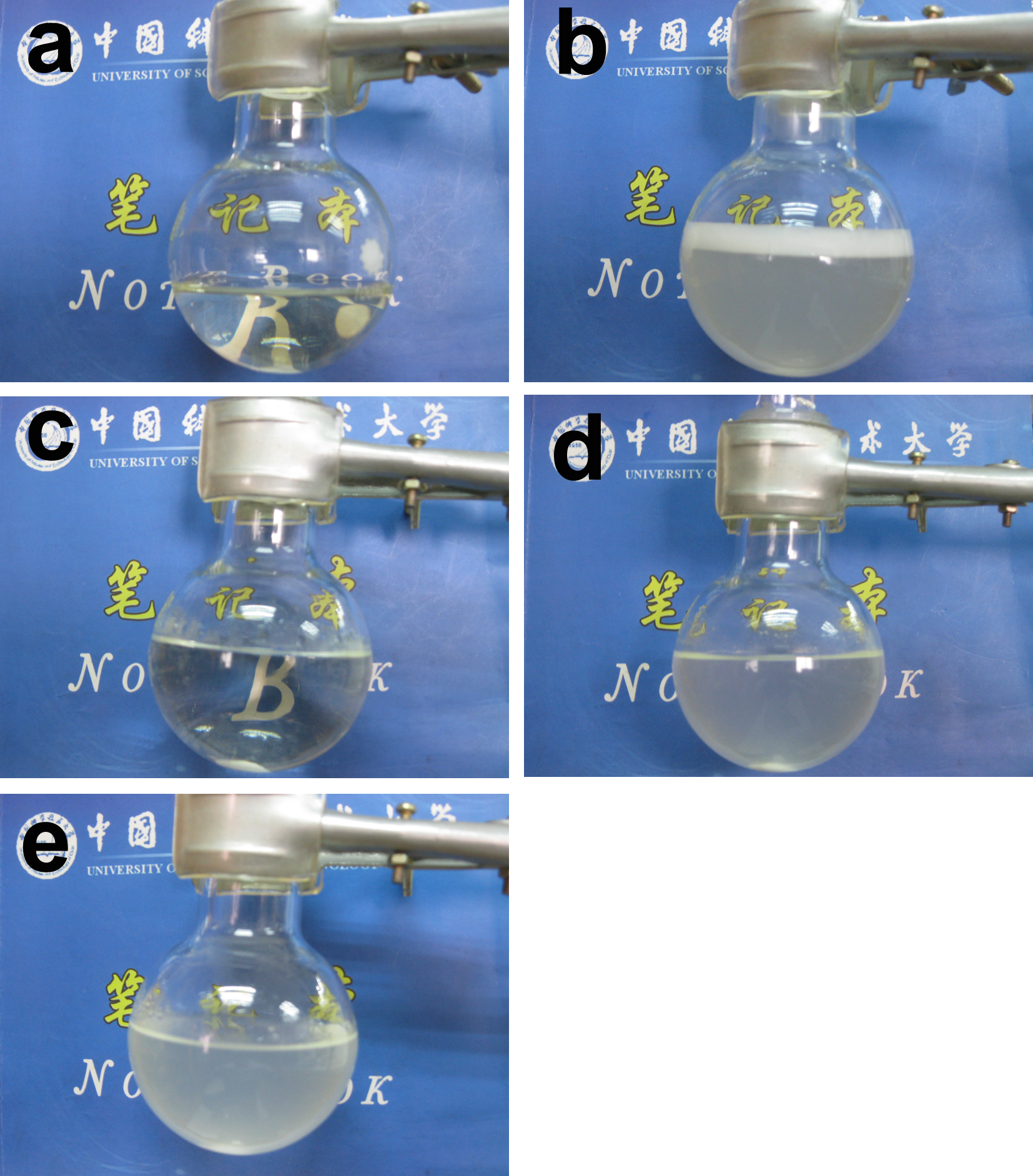


**Figure S1**

Supplement: Figure S1 — Digital pictures: (a) the clear solution after DA, TEOS and lecithin were dissolved in 30 mL of ethanol; (b) the turbid suspension obtained after a 30 mL of H2O was added into the ethanol solution; (c) the turbid suspension became clear by heating treatment in 80°C water bath for 24 hours; (d) the turbidness appeared again after the clear solution was cooled down at room temperature for 1 h, and the temperature of the suspension is close to room temperature; (e) much more turbidness was obtained after a 24 h of cooling. (DOC) [file pone.0061164.s001.doc]
