# Supplementary material for: Formation of Asymmetrical Structured Silica Controlled by a Phase Separation Process and Implication for Biosilicification
Source: PLoS One. 2013 Apr 9;8(4):e61164. doi: 10.1371/journal.pone.0061164 (PMC3621999; doi:10.1371/journal.pone.0061164)
Supplement: Table S1 — Experimental conditions and corresponding aspect ratios of the silica particles (Ethonal/water = 30∶30 vol/vol). (DOC) [file pone.0061164.s002.doc]

**Table S1**

| Sample | L0 | L1 | L2 | L3 | L4 | L5 | L6 |
| --- | --- | --- | --- | --- | --- | --- | --- |
| Initial pH | 11.6 | 11.6 | 11.6 | 11.6 | 11.6 | 11.6 | 11.6 |
| [phospholipid]  (g/L) | 0.00 | 0.35 | 0.70 | 1.00 | 1.35 | 1.70 | 2.00 |
| [DA] (mM) | 14.4 | 14.4 | 14.4 | 14.4 | 14.4 | 14.4 | 14.4 |
| Aspect Ratio  (D/T) | Flower-like spheres | 1.00 | 1.15 ± 0.02 | 1.22 ± 0.03 | 1.41 ± 0.03 | 1.60 ± 0.06 | Connected particles |
|  |  |  |  |  |  |  |  |
| Sample | D0 | D1 | D2(L5) | D3 | D4 | D5 | D6 |
| Initial pH | 11.6 | 11.6 | 11.6 | 11.6 | 11.6 | 11.6 | 11.6 |
| [phospholipid]  (g/L) | 1.70 | 1.70 | 1.70 | 1.70 | 1.70 | 1.70 | 1.70 |
| [DA] (mM) | 0.0 | 4.8 | 14.4 | 16.8 | 19.2 | 24.0 | 28.8 |
| Aspect Ratio  (D/T) | films | 1.85 ± 0.12 | 1.60 ± 0.06 | 1.54 ± 0.09 | 1.46 ± 0.07 | 1.00 | 1.00 |
